# Supplementary material for: Evaluating the Impact of the COVID-19 Pandemic on New Cancer Diagnoses and Oncology Care in Manitoba
Source: Curr Oncol. 2021 Aug 12;28(4):3081–90. doi: 10.3390/curroncol28040269 (PMC8395519; doi:10.3390/curroncol28040269)
Supplement: Supplementary file 1 [file curroncol-28-00269-s001.zip › curroncol-1277345-supplementary.pdf]

Supplementary Materials:

Evaluating the Impact of the COVID-19 Pandemic on New Cancer Diagnoses and Oncology Care in Manitoba

Kathleen M. Decker, Pascal Lambert, Allison Feely, Oliver Bucher, Julian O. Kim, Pamela Hebbard, Maclean Thiessen, Tunji Fatoye, Marshall Pitz, Rashmi Koul, and Piotr Czaykowski

Table S1. Linear regression results.

			Estimate (95% Confidence Interval)	p-Value
**Cancer diagnoses**	Intercept		543.50 (509.62–577.37)	<0.001
	COVID-19		-1946.94 (-3211.06– -682.81)	0.003
	Time		0.94 (0.41–1.47)	0.001
	Month	′	6.24 (-49.20–61.69)	0.003
		′′	59.24 (0.35–118.14)	
		′′′	-87.40 (-143.11– -31.69)	
		′′′′	57.87 (9.28–106.47)	
		′′′′′	-53.19 (-131.65–25.27)	
		′′′′′′	-1.22 (-41.59–39.15)	
	COVID-19:Time		28.27 (9.27–47.26)	0.004
**Pathology reports**	Intercept		2753.21 (2579.25–2927.18)	<0.001
	COVID-19		-7548.30 (-11233.57– -3863.03)	<0.001
	Time		3.89 (1.14–6.64)	0.006
	Month	′	141.04 (-135.09–417.17)	<0.001
		′′	561.53 (254.00–869.06)	
		′′′	-355.44 (-641.46– -69.42)	
		′′′′	487.20 (241.25–733.16)	
		′′′′′	109.76 (-300.74–524.27)	
		′′′′′′	161.57 (-40.28–363.41)	
	COVID-19:Time		107.84 (53.69–161.98)	<0.001
**Surgery**	Intercept		1216.39 (1142.11–1290.68)	<0.001
	COVID-19		-739033.58 (-1548491.14–70423.98)	0.073
	Time	′	12.97 (-101.34–127.28)	0.416
		′′	59.38 (-32.33–151.08)	
	Month	′	133.31 (16.91–249.70)	<0.001
		′′	252.81 (127.72–377.90)	
		′′′	-237.36 (-351.52– -123.19)	
		′′′′	445.91 (346.89–544.92)	
		′′′′′	-11.15 (-170.98–148.68)	
		′′′′′′	70.80 (-11.46–153.05)	
	COVID-19:Time	′	1237521.19 (-121931.90–2596974.28)	<0.001
		′′	406433.82 (-36855.86–849723.50)	
**Intravenous chemotherapy**	Intercept		1724.45 (1616.55–1832.35)	<0.001
	COVID-19		13.80 (-153.91–181.50)	0.870
	Time	′	108.85 (-17.16–234.86)	0.325
		′′	-87.46 (-215.45–40.53)	
		′′′	-81.84 (-381.84–218.17)	
		′′′′	1.14 (-246.19–248.47)	
	Month	′	64.30 (-82.13–210.72)	0.308
		′′	-49.26 (-130.39–31.88)	
**Radiotherapy treatment (first)**	Intercept		338.68 (310.72–366.64)	<0.001

	COVID-19		-15.47 (-54.16–23.23)	0.428
	Time	′	9.87 (-19.08–38.81)	0.024
		′′	22.58 (-6.91–52.07)	
		′′′	3.91 (-65.06–72.88)	
		′′′′	42.89 (-14.24–100.03)	
	Month	′	-38.00 (-74.59– -1.41)	<0.001
		′′	40.13 (2.36–77.90)	
		′′′	-57.04 (-93.63– -20.45)	
		′′′′	56.00 (24.52–87.47)	
		′′′′′	-32.10 (-82.38–18.19)	
		′′′′′′	-52.31 (-78.43– -26.18)	
**Radiotherapy fractions**	Intercept		3880.59 (3607.31–4153.88)	<0.001
	COVID-19		-563.19 (-816.57– -309.81)	<0.001
	Time		0.95 (-3.49–5.38)	0.671
	Month	′	76.12 (-368.47–520.71)	0.002
		′′	788.62 (327.16–1250.08)	
		′′′	-59.70 (-501.93–382.53)	
		′′′′	611.19 (228.08–994.30)	
		′′′′′	292.19 (-321.76–906.15)	
		′′′′′′	-108.16 (-425.12–208.81)	
**Urgent cancer care clinic visits**	Intercept		200.67 (188.38–212.97)	<0.001
	COVID-19		-32.94 (-45.64– -20.24)	<0.001
	Time		-0.32 (-0.54– -0.10)	0.006
	Month	′	12.52 (-2.83–27.87)	0.193
		′′	-1.20 (-26.23–23.84)	
		′′′	-7.86 (-19.04–3.32)	
**In-person visits**	Intercept		9249.00 (8283.53–10214.48)	<0.001
	COVID-19		-5606.68 (-6701.30– -4512.06)	<0.001
	Time		65.47 (-8.51–139.44)	0.079
	Month	′	2244.09 (1101.43–3386.75)	0.006
		′′	59.47 (-1724.81–1843.74)	
		′′′	-28.30 (-791.86–735.25)	
**All visits**	Intercept		9464.62 (8624.16–10305.08)	<0.001
	COVID-19		-72.25 (-983.14–838.65)	0.868
	Time		66.37 (4.92–127.82)	0.036
	Month	′	5.28 (-1212.12–1222.69)	0.061
		′′	1218.44 (-1.46–2438.34)	
		′′′	-863.17 (-2205.27–478.94)	
		′′′′	1729.38 (664.35–2794.40)	
		′′′′′	225.08 (-1690.60–2140.76)	
		′′′′′′	-336.50 (-1251.96–578.96)	

Abbreviations: ′, splines.

Table S2. Ratios and 95% confidence intervals (CI) between predicted and expected values.

	Ratio (95% CI)			
**Outcome**	**Average**	**Apr 2020**	**Sep 2020**	**Dec 2020**
**Cancer diagnoses***		0.77 (0.67–0.87)	1.00 (0.90–1.11)	
**Pathology reports***		0.79 (0.70–0.88)		1.07 (0.98–1.16)
**Surgery***		0.57 (0.45–0.70)	0.99 (0.88–1.11)	
**IV chemotherapy**	1.01 (0.91–1.11)			
**RT treatment (first)**	0.96 (0.85–1.06)			
**RT fractions**	0.87 (0.81–0.93)			
**Urgent Cancer Care clinic (UCC) visits**	0.82 (0.75–0.89)			
**In-person visits**	0.48 (0.38–0.58)			
**All visits**	0.99 (0.91–1.08)			

Abbreviations: CI, confidence interval; RT, radiotherapy; IV, intravenous.

* Significant COVID-19 by time interaction.
